# The Week's Work

**Published:** 1910-11-12

**Authors:** 


### The Week's Work.

### THE JURY "DO NOT APPRECIATE."

A very tangled story was unfolded in the Coroner's Court at Battersea last week, the published reports of which leave several important points obscure. An infant of ten months was observed to place a piece of coal in his mouth, and was immediately taken acutely ill. An uncle, who was present, rushed off with the baby to obtain assistance, but was overcome by exhaustion before reaching the Battersea Anti-Vivisection Hospital, and handed the child to a neighbour, who took it to that institution. It does not transpire whether the neighbour was told the history of what had happened, though it may be presumed that this was done. The resident medical officer at the Anti-Vivisection Hospital could detect no foreign body, though afterwards it turned out that a piece of coal an inch square was jammed in the larynx; according to one report in the Press he diagnosed pneumonia, and according to another he made no diagnosis, but ordered a hot bath and a mustard poultice. He instructed the sister to telephone to the Wandsworth Infirmary; this was done, and the message given was that the case required instant tracheotomy. The Anti-Vivisection medical officer denies that he authorised the message about tracheotomy; but if he failed to notice the pressing need for this measure, then the sister at any rate did, and had evidently little faith in mustard-poultice treatment. To send away an infant suffering from acute dyspnœa with a history of "swallowed" coal is an act which the medical profession as well as the jury "do not appreciate"; to miss the diagnosis is bad enough, to omit treatment is too bad. At all events, the child was admitted to the infirmary at the point of death, but was resuscitated by the immediate performance of tracheotomy in the receiving-room kitchen; it developed pneumonia, however, and died three days later. After death the piece of coal was found in the larynx. Whether the Anti-Vivisection doctor recognised the urgency of tracheotomy but lacked courage to perform it, or whether he failed to recognise it, the result is equally humiliating and disastrous; and it is not surprising that the foreman added: "The jury do not appreciate the action of the Anti-Vivisection Hospital."

### HOSPITALS AND EXPERT ASSISTANCE.

Anyone who had the opportunity of reading Mr. W. G. Carnt's report on the Chesterfield and North Derbyshire extensions (for that is what his letter to Mr. Barnes, referred to on p. 181 of our last issue, amounts to) would agree that the hospital was very wise in calling in expert advice before it decided in what way to attack its difficulties. The question of reconstruction at Chesterfield five years ago was difficult and thorny. The only alternative was demolition, which meant an expenditure of about £60,000. It was owing to these difficulties that Mr. Carnt was called in. Here we may remind our readers of an article on "The Practice of Hospital Administration," published in The Hospital of November 27, 1909, where we "counselled the authorities everywhere who have new hospitals to build, if they employ an architect assessor, to associate with him someone who can have no interest whatever in making the award, except to the best man, and who is known to possess an intimate knowledge of hospital administration in all departments." This counsel is equally suggestive where no assessor is employed. It is not merely that Mr. Carnt is a man of wide experience, having built or helped in building two large and several smaller institutions, and being an active superintendent himself, though that, of course, is a fact of the greatest weight. It is rather that the impartiality of experience acquired in other institutions can settle difficulties which, if discussed locally, without such external influence, often prove adamantine. Where such expert assistance is given under the voluntary system its results are particularly fruitful. Voluntary hospitals learn to depend on one another, and individuals of experience and authority gain a statesmanlike grasp of the voluntary hospitals as a whole, which is invaluable in the administration of their own local institutions. There is no doubt, too, that a friendly critic, coming from another part of the hospital world, can save an institution from many blunders. Hospital extension and building have become such great problems nowadays that oppressed, as they are, with the difficulty of raising money for the scheme, the local hospital authorities can hardly grasp the other aspects of their problem with an open mind. But it is just to these general questions that an authority from elsewhere brings the clearness of vision that is wanted. He is not depressed at the very start by knowledge of the difficulties attending an appeal, which sometimes make the best schemes look gloomy to the men of the neighbourhood. From all this he is free, and this is what makes his advice so valuable. The Chesterfield and North Derbyshire Hospital is an instance of this, which is worth reflecting on.

### THIS WEEK'S DRUG MARKET.

Few noteworthy alterations in the prices of drugs have occurred since the last report appeared. There has been a further small advance in the price of cream of tartar; as mentioned last week, the cause of the steady increases in the value of this product, and of products dependent upon cream of tartar for their manufacture, is the failure of the wine crop. The advanced rates are likely to be in force for some time to come, and there appears to be little advantage in delaying purchases. Gum benzoin continues to fetch good prices. Valerian rhizome is scarce and dearer. Oil of cubebs is tending lower in price. Oil of cloves and oil of aniseed are slightly dearer. Ergot of rye is still tending higher in value. Coca leaves are dearer. Low prices are quoted for carbolic acid. Morphine and codeine are unchanged in price. Business generally has been rather quieter during the week, but there are signs of an impending improvement.

